# Oxygen‐Bridged Cobalt–Chromium Atomic Pair in MOF‐Derived Cobalt Phosphide Networks as Efficient Active Sites Enabling Synergistic Electrocatalytic Water Splitting in Alkaline Media

**DOI:** 10.1002/advs.202306678

**Published:** 2023-11-23

**Authors:** Zepeng Lv, Huakui Zhang, Chenhui Liu, Shaolong Li, Jianxun Song, Jilin He

**Affiliations:** ^1^ Zhongyuan critical metals laboratory Zhengzhou University Zhengzhou Henan 450001 P. R. China; ^2^ Henan province industrial technology research institute of resources and materials Zhengzhou University Zhengzhou Henan 450001 P. R. China

**Keywords:** bifunctional electrocatalysts, electrocatalysis, metal‐organic frameworks, synthesis, water splitting

## Abstract

Electrochemical water splitting offers a most promising pathway for “green hydrogen” generation. Even so, it remains a struggle to improve the electrocatalytic performance of non‐noble metal catalysts, especially bifunctional electrocatalysts. Herein, aiming to accelerate the hydrogen and oxygen evolution reactions, an oxygen‐bridged cobalt–chromium (Co‐O‐Cr) dual‐sites catalyst anchored on cobalt phosphide synthesized through MOF‐mediation are proposed. By utilizing the filling characteristics of 3d orbitals and modulated local electronic structure of the catalytic active site, the well‐designed catalyst requires only an external voltage of 1.53 V to deliver the current density of 20 mA cm^−2^ during the process of water splitting apart from the superb HER and OER activity with a low overpotential of 87 and 203 mV at a current density of 10 mA cm^−2^, respectively. Moreover, density functional theory (DFT) calculations are utilized to unravel mechanistic investigations, including the accelerated adsorption and dissociation process of H_2_O on the Co‐O‐Cr moiety surface, the down‐shifted d‐band center, a lowered energy barrier for the OER and so on. This work offers a design direction for optimizing catalytic activity toward energy conversion.

## Graphical abstract

An effective strategy is suggested to greatly strengthen alkaline bifunctional activity and stability of MOF‐derived Co_2_P catalyst by introducing the oxygen‐bridged trivalent Cr, achieving directional and accelerated adsorption and dissociation of water molecules and a lowered energy barrier for the OER on oxygen‐bridged Cr‐Co (Co‐O‐Cr) collaborative coordination with strong electron coupling.

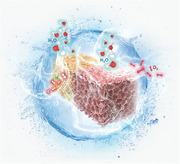

## 1 Introduction

Metal‐organic frameworks (MOFs), also tagged as porous coordination polymers (PCPs), possess periodic inorganic metal ions/clusters‐organic ligands sub‐structure assembled by the coordination reaction,^[^ ^1^, ^2^ ^]^ which show great application prospects in energy storage, biomedicine, catalysis etc., owing to the characteristic of adjustable pore size, large specific surface area, tunable structures.^[^ ^1^, ^3^, ^4^ ^]^ Over the past 20 years, catalytic field became the first proposed and the fastest‐growing application of MOF materials,^[^ ^5^, ^6^, ^7^ ^]^ and it had been particularly attracting an increasing amount of attention as catalysts for electrochemical water splitting, which contained electrochemical oxygen evolution reaction (OER) and hydrogen evolution reaction (HER).^[^ ^8^ ^]^ However, even if thousands of MOF structure have been discovered now, few of them can be used for electrocatalysis directly due to the limitation of their poor electrical conductivity, saturated active sites, poor stability, and so on.^[^ ^4^, ^9^ ^]^ In particular, the metal ions in MOFs are almost completely coordinated with organic linkers, thus there are no extra active sites or redundant empty orbitals for the combination with reactant intermediates, which makes the progress of catalytic reactions reach a bottleneck.^[^ ^9^ ^]^

Currently, there exist some intriguing optimization strategies to negotiate the overall water splitting (OWS) efficiently for MOFs. One feasible strategy to enhance the electrocatalytic activity is to use MOFs as ideal precursors for the synthesis of MOF‐derivatives (e.g., transition‐metal layered double hydroxides, oxides, sulfides, carbides, nitride).^[^ ^1^, ^3^, ^9^ ^]^ Particularly, atomically dispersed metal ions in MOFs can guarantee a satisfactory distribution of MOF‐derived metallic compounds.^[^ ^3^ ^]^ Hence in recent years, some investigators utilized the atomic‐level uniform dispersion characteristic of MOFs to remove the linkers homogeneously by an in situ etching process, preparing the ultrathin 2D transition metal hydroxides or mono/polyoxometalates through the interconnection between free metal parts and hydroxide/oxometallate radicals.^[^ ^4^, ^10^, ^11^ ^]^ Zhang et al. synthesized a type of ultrathin transition metal hydroxide by in situ etching of a Co‐MOF, which showed an attractive electrocatalytic activity with a low overpotential of 223 mV to reach 10 mA cm^−2^ in alkaline OER process.^[^ ^4^ ^]^ Similarly, NiCoFe‐based trimetallic MOF nanostructures with foam‐like architecture was reported by Qian et al., which delivers a minimum overpotential of 257 mV at 10 mA cm^−2^.^[^ ^12^ ^]^ Homogeneous work for OER also includes Co‐LDH@MOF,^[^ ^13^ ^]^ alkali‐etched Ni(II)‐based MOF,^[^ ^14^ ^]^ Fe–Ni LDH/MOF,^[^ ^15^ ^]^ and so on.

Although their multi‐level nanoarrays have significant advantages in catalytic reactions, it is obvious that these catalysts are far from being optimized for practical application requirements, especially only one half‐reaction of OER can be achieved. Integrating both the merits of HER and OER in a single bifunctional MOF‐derived catalyst still remain a challenge for the mesoscale design of catalysts. In this regard, constructing a MOF‐derived catalyst with advantageous composition, morphology, and bifunctional catalytic activity is urgently needed to promote the application of MOF‐based materials in the field of electrochemical water splitting and the development of hydrogen production. Moreover, most known MOFs or MOF‐derivatives are in the form of powders, which present significant inferior performance compared with the catalyst grown on porous frameworks (e.g., foamed nickel). In especial, nickel foam (NF) as a typical representative among them not only enriched electrochemically active sites, but also promoted the elimination of bubbles generated by HER/OER and the ion exchange at the interface of multiphase reactions.^[^ ^8^, ^16^, ^17^, ^18^ ^]^ Moreover, the prepared self‐supporting catalyst on NF can greatly the deterioration of the intrinsic activity and electrical conductivity caused by the introduction of binder (e.g., Nafion) in traditional processes.

In this work, we, therefore, developed a facile strategy to fabricate hierarchically structured transition metal phosphides with a low degree of crystallinity on 3D conductive NF by successive room temperature solution‐phase growth, in situ etching and phosphorization approach, where oxygen atoms bridged cobalt and chromium metal atoms to form the atomically dispersed active moiety (Co‐O‐Cr) anchored on the MOF‐derived phosphor‐nitrogenated porous carbon (named Cr‐Co‐P). As expected, this well‐manipulated catalyst exhibits much enhanced electrocatalytic activity for both HER and OER, owing to the multilevel open structure, optimized active site and 3D conductive NF substrate, and the materials has been employed in overall water splitting reaction with an impressive performance.

## 2 Results and Discussion

**Figure 1:**
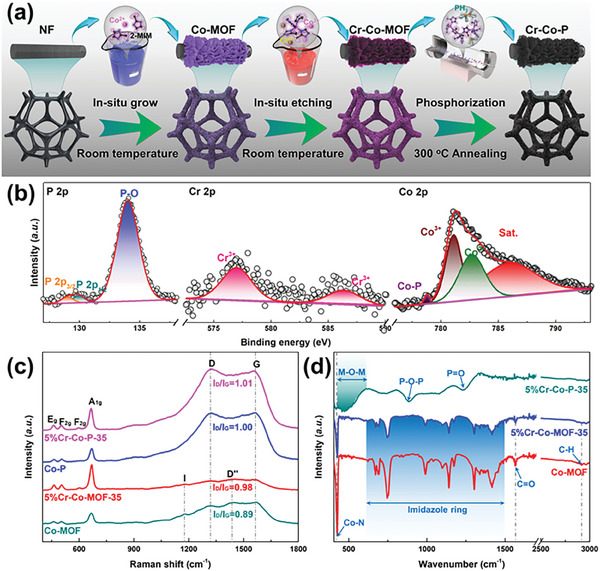
a) The scheme of the formation mechanism of Co‐MOF derived Cr‐Co‐P composite, b) XPS high‐resolution, c) Raman, and d) FT‐IR spectra of samples.

The key segments of our facile strategy are schematically illustrated in **Figure** **1**a (see details in the Experimental Section, Supporting Information). The Co‐MOF precursor is synthesized by a previously reported method.^[^ ^4^, ^11^, ^16^, ^19^ ^]^ First, a piece of clean NF was successively immersed into an aqueous solution containing 2‐methylimidazole (2‐MIM) and a mixed solution of 2‐MIM and Co(NO_3_)_2_ for 6 h each without stirring, and well‐aligned Co‐MOF nanosheets can be synthesized on the 3D NF skeleton successfully, which changed the color of NF from bright silver to purple. This obtained Co‐MOF nanoarrays were initially characterized by field‐emission scanning electron microscopy (FESEM) and displayed the homogeneous spindle‐type shape with a smooth surface (Figure S1a, Supporting Information). The surface of as‐prepared Co‐MOFs were subsequently beginning to be reconstructed by an in situ etching process using cobalt chromium mixed ethanolic solution (named Cr‐Co‐MOF), where we first studied the synthesis of monometallic Co(OH)_2_ nanosheets and investigated the effect of etching time on catalyst morphology. Specifically, the shorter etching time can only rise the surface roughness of Co‐MOFs as depicted in Figure S1b (Supporting Information), and it is difficult to support the completion of surface reconstruction, resulting the restricted specific surface area and number of active sites. While, the longer etching time will make the MOF structure collapse of well‐aligned arrays, which is also unfavorable for the catalytic reaction (Figure S1e, Supporting Information). Because transition‐metal hydroxide trends to form an amorphous sheet‐like structure, Co‐MOF converted through in situ etching possesses a hierarchical 2D leaf‐like structural unit (Figure S1c–d, Supporting Information), where the unique microstructure is still maintained after the introduction of Cr and present a more dominant secondary array with plentiful mesoporous (Figure S1f, Supporting Information). Subsequently, it was converted into Cr‐doped cobalt phosphide networks and MOF‐derived phosphor‐nitrogenated porous carbon (named as Cr‐Co‐P) via a typical phosphorization process, which clearly perfectly inherited the unique structure of the precursor (Figure S2, Supporting Information).

The crystal structure and crystallinity of the as‐synthesized catalysts were confirmed by X‐ray diffraction (XRD). As shown in Figure S3a (Supporting Information), the resultant XRD patterns only identify three strong peaks between 40° and 80° corresponding to metallic Ni substrate (PDF No.04‐0850) and a weak peak corresponding to Co_2_P (PDF No.32‐0306) by matching with that of the simulated Co‐MOF and other standard cards. One reason is that MOFs can degrade numerous beneficial properties under external physical or chemical stimuli (e.g., etching, annealing treatments), including crystallinity.^[^ ^20^ ^]^ Another reason is that weak X radial diffraction signal of loaded active substance is covered up by robust signal of NF, which has also been mentioned in other studies.^[^ ^8^, ^21^ ^]^ In addition, the Co‐MOF powder sample peeled off from NF was were further characterized, and the results are shown in the following Figure S3b (Supporting Information). It is obvious that the diffraction peak of the powder product matches well with ZIF‐L‐Co (simulated) reported by Zhang et al,^[^ ^22^ ^]^ indicating that the product synthesized through this strategy is indeed Co‐MOF. To further investigate the evolution law of elements and phases, X‐ray photoelectron spectroscopy (XPS) analysis was performed. As displayed in Figure S3b (Supporting Information), Co‐MOF is composed of C, N, O, and Co. Specifically, the C 1s spectrum of Figure S4a (Supporting Information) can be divided in to four peaks at 284.5, 285.8, 286.9, and 288.3 eV, respectively, corresponding to C═C, C═O, C═N, and ─COOH.^[^ ^23^, ^24^, ^25^ ^]^ In the N 1s of spectrum of Figure S4b (Supporting Information), the peaks at 397.9, 400.2, 398.9, 401.6, and 406.4 eV present the Pyridinic N, Pyrrolic N, C─N, Graphitic N and trace of Chemisorbed N from the Co‐2MIM group,^[^ ^24^ ^]^ which demonstrate the coexistence of Co‐MOF. Due to the influence of synthesis process in aqueous medium, the O 1s spectrum contains two peaks of 531.2, 532.7 eV, which point to metal hydroxides (M‐OH) and adsorbed O.^[^ ^8^, ^17^ ^]^ The hydrolysis‐controlled in situ etching process makes the signal of M‐OH and adsorbed O enhance and introduces the chromium element in the form of Cr^3+^, which shows the presence of Cr 2p_3/2_ and Cr 2p_1/2_ pair at 576.8 and 586.2 eV of high‐resolution Cr 2p spectra (Figure S4d, Supporting Information).^[^ ^26^ ^]^ Although there is some noise in the spectrum of chromium due to the low element content, it still does not affect the judgment of the occurrence state of the Cr elemental. Here, it was found that there was no signal of Ni 2p in the XPS results of all samples in this study by comparing with the control group previously reported by us,^[^ ^18^ ^]^ which indirectly confirmed that no dissolved nickel element was introduced into the catalyst. After phosphating treatment, the signal of P 2p appears and accompanies and is accompanied by a weakening of the peaks of C 1s, N 1s, and so on. Where, the P 2p spectrum can be fitted into three peaks (Figure 1b), with the main P‐O peak at 134.1 eV and a pair of relatively weak peaks of P 2p_3/2_, P 2p_1/2_ at 129 and 130 eV.^[^ ^8^, ^18^ ^]^ The doped N in porous carbon is primarily in the main form of Pyrrolic N (Figure S4b, Supporting Information), and the characteristic signals related to in Co‐N_x_ moieties originating from Co‐MOF weaken (Co‐N, Pyridinic N) or even disappear (Chemisorbed N) with the strengthening of the C═N signal in high‐resolution C 1s spectra (Figure S4a, Supporting Information), implying the conversion from MOF to N doped carbon. The post‐treatment methods for MOFs have rapidly advanced, in which porous carbons is one of the common products after thermal treatment of MOFs. At the pyrolysis process, the ligands will decompose and transform to porous carbon or carbon doped with heteroatom. It is widely known that porous carbon with extraordinary electrical, mechanical, and thermal properties not only avoids the problem of easy dissolution of MOFs materials in alkaline media, but also increases the conductivity of the whole catalyst, which promotes the rapid migration of electrons in the catalyst.^[^ ^27^ ^]^ Second, as a porous framework, it plays a supporting and dispersing role in the sheet‐like catalyst, resulting in the exposure of abundant catalytic active centers. And as one of the components of heterogeneous catalysts, it forms strong interactions with Co_2_P to optimize and adjust the electronic structure of Co_2_P, which has been confirmed in the theoretical calculation section. However, due to extensive research reports on the role of porous carbon in MOFs derivatives, this article did not elaborate on it in detail in subsequent discussions.In the Co 2p_3/2_ spectrum (Figure 1d), four signals have appeared in the binding energy of Co, including Co‐P, Co^3+^, Co^2+^ and a shakeup satellite (Sat.) at 778.7, 780.9, 782.5, and 785.9 eV respectively, where the lower binding energy of Co‐P indicates the presence of Co^n+^ with valence state *n* < 2 in Co 2p, thereby confirming that the reduction of Co atoms on the surface of catalysts.^[^ ^4^, ^8^, ^18^, ^28^ ^]^

Especially, the peaks of Co 2p_3/2_ shift to a high energy level by 1.5 eV, after incorporating Cr element in to Co_2_P, indicating the decreased electron density in Co_2_P. This strongly verifies that some Co elements Co_2_P were tightly bound to Cr. Additionally, it can be noted that the valence of Cr in Cr‐Co‐P is still tervalence without Cr═P bond evidently, indicating that the surface Cr^3+^ is in hybrid species of CrO_x_. In the Raman spectra of samples (Figure 1c), the signal peaks of Co elements can be attributed to the vibration modes of E_g_ (461.5 cm^−1^), F_2g_ (503.9, 596.5 cm^−1^), and A_1g_ (665.9 cm^−1^). For Co‐MOF, there were four characteristic peaks located at 1565, 1437, 1318, and 1179 cm^−1^, typically belonging to G, D″, D, and I bands of carbon, respectively.^[^ ^29^, ^30^ ^]^ Among them, the D‐band mainly comes from the stretching vibration of the C─C bond and C═C double bond, and its strength reflects the proportion of defects, structural distortion, and amorphous parts in the sample. The G‐band mainly comes from the stretching vibration of sp2 hybrid carbon atoms and π electrons in the material, and its strength reflects the layer number and lattice integrity of the material. Usually, the ratio of two Raman bands of I_D_/I_G_ was selected to reflect the disorder degree of carbon materials. We obtained I_D_/I_G_ values of 0.89, 0.98, 1.00, and 1.01 for Co‐MOF, Cr‐Co‐MOF, Co‐P, and Cr‐Co‐P, respectively. Obviously, after undergoing annealing and converting to porous carbon, the low degree of graphitization and high degree of defects in the catalysts were generated, which had been confirmed by previous studies that carbon materials containing defects exhibit excellent performance in various electrochemical reactions.^[^ ^31^ ^]^ At the same time, the transformation process from MOF to carbon materials was further validated in FT‐IR analysis. As shown in Figure 1d, the different peaks of Co‐MOF in 600–1500 cm^−1^ are attributed to the stretching and bending modes of the imidazole ring, and the peaks at 424, 1565, and 2920 cm^−1^ correspond to the Co‐N stretching mode, C═O and stretching vibration of C─H single bond of Co‐MOF.^[^ ^32^, ^33^ ^]^ Interestingly, these characteristic peaks related to Co‐MOF significantly weaken after etching treatment and disappear after phosphating treatment, implying that MOF material has undergone transformation. The final product only exhibits characteristic peaks corresponding to stretching of M─O─M bonds, P═O stretching and P─O─P bonding at 400–600, 1229, and 885 cm^−1^, respectively.^[^ ^23^, ^34^, ^35^ ^]^

**Figure 2:**
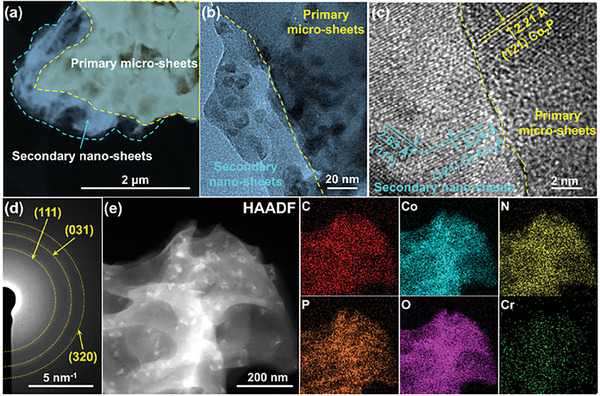
a,b) Transmission electron microscope (TEM), c) high‐resolution transmission electron microscope (HRTEM) images, and d) selected area electron diffraction (SAED) pattern of 5%Cr‐Co‐P‐35, e) Elemental mapping showing the uniform distribution of C, N, Co, Cr, P, and O elements in 5%Cr‐Co‐P‐35.

Transmission electron microscope (TEM) is further used to analyze the crystal structure and elemental composition of the final sample Cr‐Co‐P. As displayed in **Figure** **2**a,b, the Cr‐Co‐P exhibits an obvious multi‐level structure with a line of demarcation between them. The primary micro‐sheets are consisted of numerous ultrafine crystalline nanoparticles with average particle size of 15 nm and amorphous carbon components (Figure 2c), where distinct crystal spacing of 2.21 Å on the nanoparticles can be identified in the HRTEM image, corresponding to the (121) facets of Co_2_P. Moreover, the specific feature of secondary nano‐sheets presents a slightly lattice expansion according to the crystal spacing of (121) facets, which is caused by differences in radii between cobalt and chromium ions.^[^ ^36^, ^37^ ^]^ Likely, the (111), (031), (320) facets of Co_2_P are also be indexed according to the analysis of SAED. The presence and uniform distribution of C, Co, N, P, O, and Cr elements in Cr‐Co‐P is visualized by energy dispersive spectrometer (EDS) elemental mapping, indicating that Cr element is uniformly doped in Co_2_P anchored on porous carbon. According to the EDS result, the atomic percentages of Co and Cr in 5%Cr‐Co‐P‐35 are actually 6.52% and 0.11%, respectively. The above characterization results indicate that Cr doped Co_2_P are successfully prepared with multilevel open nanosheet arrays.

**Figure 3:**
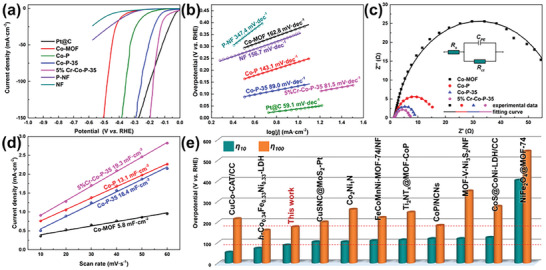
a) HER polarization curves, b) HER Tafel slopes (measured according to the HER performance in (a)), c) Nyquist plots of different catalysts, d) Electrochemical double‐layer capacitances for different catalysts, e) comparison between 5%Cr‐Co‐P‐35, and other MOF‐derived catalysts.

The activity of the Cr‐Co‐P catalyst toward electrochemical water splitting was assessed in argon‐saturated 1.0 M KOH by using a polarization curve at a scan rate of 5 mV s^−1^. In order to evaluate the effect of in situ etching conditions (e.g., etching time, chromium content) on electrocatalytic activity, catalyst samples obtained under various conditions were first subjected to a simple HER reaction test (Figure S5, Supporting Information). Clearly, the sample with the highest HER activity was obtained by 35 min etching and introducing Cr element of 5% molar percentage (named 5%Cr‐Co‐P‐35). There is generally no positive correlation between catalyst activity and doping element content, and instead it presents a volcanic type.^[^ ^38^, ^39^ ^]^ When the Cr content in the catalyst is too low, the activity of the catalyst is limited due to the lack of Cr O‐Co active sites on the surface. But as the Cr^3+^ content in the etching solution increases to very high concentration, there is an impurity phase with mainly Cr element in the form of nanosheet (Figure S6, Supporting Information). Especially, Cr‐LDH has poor catalytic performance. Hence, the decrease of catalytic activity for the samples with high Cr/Co ratios possibly resulted from the active site block by the surface‐covered impurities. After co‐constructing multilevel open nanosheet arrays, the optimized 5%Cr‐Co‐P‐35 exhibits remarkable catalytic activity with overpotentials of 87 and 176 mV to reach a current density of −10 and −100 mA cm^−2^ (*η*_−10_= 87 mV, *η*_−100_= 176 mV), which is superior to those of NF (*η*_−10_= 478 mV), P‐NF (phosphated NF, *η*_−10_= 355 mV), Co‐MOF (375, 478 mV), Co‐P (235, 356 mV) and Co‐P‐35 (134, 254 mV). Even, such an excellent electrocatalytic activity makes 5%Cr‐Co‐P‐35 much better than commercial Pt@C catalysts in high current density, for example, an overpotential reduced by nearly 20 mV is obtained by 5%Cr‐Co‐P‐35 at −100 mA cm^−2^ compared with Pt@C. Notably, the process of alkaline HER is generally considered to include two microscopic steps, e.g. Volmer reaction (H_2_O + e^−^ → H_ad_ + OH^−^), Heyrovsky (H_ad_ + H_2_O + e^−^ → H_2_ + OH^−^) or Tafel (H_ad_ + H_ad_ → H_2_) reaction.^[^ ^17^, ^30^ ^]^ The adsorption and desorption kinetics of various intermediates on the catalyst surface can also affect the HER performance, therefore, the Tafel slope and electrical impedance spectroscopy (EIS) were fitted to accurately assess catalytic kinetics. **Figure** **3**b shows the Tafel plots converted based on *η* = a + b × log|*j*|, wherein only linear portions were selected to give a comparison. The minimal Tafel slope of 5%Cr‐Co‐P‐35 (81.5 mV dec^−1^) indicates the excellent electrocatalytic activity and compliance with the Volmer‐Heyrovsky mechanism. Impressively, charge transfer resistances (R_ct_) as another important indicator for evaluating catalyst activity have been characterized by EIS at −0.1 V versus RHE. As depicted in Figure 3c, both in situ etching and phosphating treatment can significantly reduce *R*_ct_ of the catalyst (see details in Table S1, Supporting Information). However, 5%Cr‐Co‐P‐35 obtained through coupling regulation of two optimization methods possesses the smallest *R*_ct_ (5.67 Ω), demonstrating its high electrochemical intrinsic activity.

**Figure 4:**
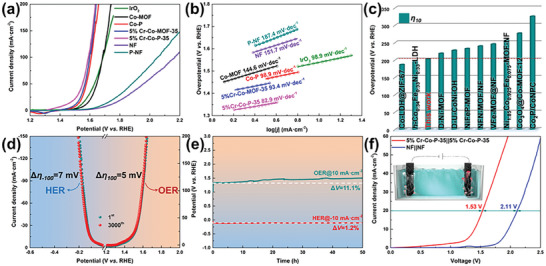
a) OER polarization curves, b) HER Tafel slopes (measured according to the OER performance in (a)), c) Comparison between 5%Cr‐Co‐P‐35 and other MOF‐derived catalysts, d) polarization curves before and after 3000 cycling testing, e) the chronopotentiometric curve of the 5%Cr‐Co‐P‐35 electrode tested at a constant current density of ± 10 mA cm^−2^ for 50 h, and f) linear sweepment voltametry of water electrolysis using 5%Cr‐Co‐P‐35 as both HER and OER catalysts in 1 m KOH.

To further understand the mechanism for the enhancing electrocatalytic activity of 5%Cr‐Co‐P‐35, a cyclic voltammetry (CV) method was investigated to measure the electrochemical double layer capacitances (C_dl_) in the non‐Faraday region (Figure S7, Supporting Information), in which the electrochemically effective surface area (ECSA) is linearly proportional to C_dl_ (ECSA = C_dl_/C_s_). As observed in Figure 3d, the C_dl_ value of 5%Cr‐Co‐P‐35 is 19.3 mF cm^−2^, larger than Co‐P (13.1 mF cm^−2^), Co‐P‐35 (16.4 mF cm^−2^) and Co‐MOF (5.8 mF cm^−2^). This phenomenon confirms that 5%Cr‐Co‐P‐35 has the largest ECSA, which has been confirmed to be beneficial for improving electrocatalytic activity through erstwhile investigations.^[^ ^16^, ^17^, ^40^ ^]^ In addition, compared with other similar MOF or MOF‐derived catalysts, 5%Cr‐Co‐P‐35 also has a competitive HER electrocatalytic activity (Figure 3e; Table S2, Supporting Information). Moreover, the bifunctional nature of the string of 5%Cr‐Co‐P‐35 fabricated electrodes was further performed for the OER properties in 1 M potassium hydroxide (KOH), where 5%Cr‐Co‐P‐35 afforded a much smaller overpotential of 203 mV at an OER current density of 10 mA cm^−2^ and a smaller Tafel slope of 82.9 mV per decade (**Figure** **4**a,b). Hence, OER catalytic process was also accelerated by constructing designed micro/nano‐structures and modified electronic structures. In general, the high‐performance OER catalysis by 5%Cr‐Co‐P‐35 was also observed to be superior than that of those catalysts toward OER performance under the same conditions (Figure 4c; Table S3, Supporting Information). Finally, the electrochemical stability of the catalyst was explored by a long‐term CV scanning of 3000 cycles. As shown in Figure 4d, the polarization curves of both HER and OER before and after the cycling test are almost overlapped under the alkaline condition, in which only 7 and 5 mV shifts can be observed after the 3000‐cycling at the current density of ± 10 mA cm^−2^. In addition, the voltage–time (*V–t*) curves are carried out to explore the long‐term durability of 5%Cr‐Co‐P‐35 at a fixed current density of ± 10 mA cm^−2^, where the *V–t* curve could maintain nearly no decay within 50 h for HER and has only a small attenuation under OER condition, owing to the generated corresponding hydroxides on the surface by in situ self‐reconstruction. Whatever, the above experimental results not only show the feasibility of bifunctional electrocatalytic applications but also excellent stability. In the process of water electrolysis, hydrogen and oxygen will form on the electrode surface, especially in the case of high current, the bubble formation speed is faster, and the bubble covering the electrode surface will not only increase the total resistance of the entire electrolysis system, but also lead to the decrease of the boundary between the electrolyte and the electrode, that is, reduce the catalytic active area. Thereby, the buildup of bubbles raises sum of overpotential needed for electrolyzed water, which is also one of the important sources of energy enhancement in electrolyzed water reaction. For the direct construction of self‐supporting electrodes in the form of porous structure, more effectively O_2_ and H_2_ diffusion and failure avoidance of actives sites can be implemented compared to the erection of electrodes using powder‐form catalysts with multiple steps. Therefore, 5%Cr‐Co‐P‐35 has potential to exploit integrated electrodes to reach excellent behavior. Considering the outstanding HER and OER performances of 5%Cr‐Co‐P‐35, a dual‐electrode electrolyzer employing the dual‐function catalyst was constructed for testing overall water splitting. Figure 4f compares the LSV for 5%Cr‐Co‐P‐35||5%Cr‐Co‐P‐35 and NF||NF. Obviously, the applied voltage of 5%Cr‐Co‐P‐35||5%Cr‐Co‐P‐35 system to afford a current density of 20 mA cm^−2^ is as low as 1.53 V, substantially lower than NF||NF (2.11 V). Such excellent electrolyzer even outperforms the most benchmark couples reported in the literature (Table S4, Supporting Information). Moreover, this excellent electrolyzer even can be able to withstand high current (> 200 mA cm^−2^) testing for > 24 h without any significant current attenuation (Figure S8, Supporting Information). By analyzing the product characterization after stability testing as shown in Figure S9 (Supporting Information), it can be concluded that the morphology of the catalyst has undergone a certain degree of change owing to surface reconstruction. Even if the presence of electrons in cathode could avoid the dissolution rate of the catalyst and surface oxidation,^[^ ^41^ ^]^ the surface compositions (≈2 nm depth) of the electrocatalyst are generally prone to modification through cathode electrochemical reconstruction,^[^ ^42^ ^]^ resulting in change of outer morphology. Hence, this also explains the reason for the appearance of nano island particles on the surface of the sample after HER testing (Figure S9a, Supporting Information). The XPS spectra (Figure S9b–d, Supporting Information) showed that the there was no significant change in Co 2p and Cr 2p, but instead the peak signal of P─O bonding in P2p decreased due to deoxidation caused by reduction. Meanwhile, electrochemical reconstruction can also be carried out on the surface of anode via quickly oxidation reaction that led to amorphized the surface and optimized OER performance.^[^ ^8^, ^18^ ^]^ Surprisingly, the outer surfaces of anodic catalyst found many hexagonal nanoflakes after stability test (Figure S9e, Supporting Information) like Zhang et al.^[^ ^4^ ^]^ mentioned. Especially, the contents of Co, Cr, and Ni present in the electrolyte (collected and analyzed after OWS catalysis) are shown in Figure S10 (Supporting Information), where the dissolution contents of Co, Cr and Ni are below 0.175, 0.046, and 0.01 µg mL^−1^, respectively. This phenomenon also indirectly confirms the occurrence of catalyst surface reconstruction, causing a decrease in Cr 2p and P 2p signals while also causing the disappearance of Co‐P, P 2p_1/2_, and P 2p_3/2_ peaks (Figure S9f–h, Supporting Information). In addition, the content of dissolved nickel in the ICP‐OES detection results is also > 0.01 µg mL^−1^, which further confirms the conclusion that nickel has not undergone significant dissolution.

**Figure 5:**
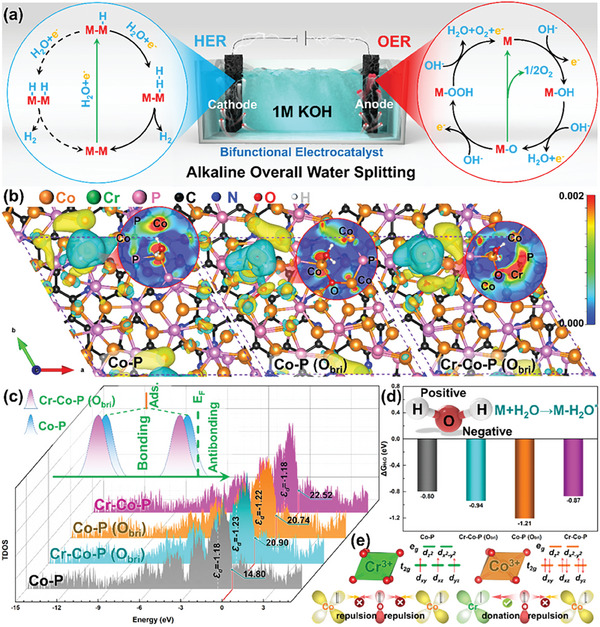
a) Schematic diagram of the alkaline overall water splitting, b) the charge density distribution of Co‐P, Co‐P (O_bri_) and Cr‐Co‐P (O_bri_) and corresponding c) DOS plots and d) free energy diagram of H_2_O on the metal centers from the basal plane (001), and e) schematic representations of the electronic coupling between Co, Cr, and O.

Open structure resulted from the MOF‐mediated etching conversion process leads to the increment of active sites, which brings extraordinary bifunctional activities compared to their pristine forms, as revealed by these experimental results. For understanding the derivation of improved activity from an atomic and electronic scale, the density functional theory (DFT) calculations were used to exam the electronic structure and free energy diagrams of electrocatalytic reaction on the MOF‐derived basal plane. We constructed the crystal structure of MOF‐derived Co_2_P, together with the catalyst introducing Cr‐O‐Co dispersed active moiety on the surface as reference (Figure S11, Supporting Information). Here, although the slab models may not be fully representative of the experimental materials, they provide insights for theoretical mechanism analysis. Whatever, the DFT geometry optimization indicates that the interfacial C, P and N atoms on the MOF‐derived porous carbon materials side connect with the Co atoms on the cobalt phosphide side by the strong Co─C, Co─N, and Co─P bonds and thereby cause interface electron rearrangement and strong electron coupling as shown in Figures S11 and S12a (Supporting Information). As is known, the HER pathway in alkaline media follows either the Volmer‐Heyrovský or Volmer‐Tafel mechanisms, where proton (H^+^) scarcity in the electrolyte makes it difficult to directly obtain protons by Volmer and Heyrovský steps in alkaline and neutral conditions. Under such circumstances, H_2_O molecules undoubtedly become the unique proton donor choice, and the specific mechanism of Volmer and Heyrovský steps is shown as follows: H_2_O + e^−^ + * →*H_ads_ + OH^−^ (Volmer step), *H_ads_ + H_2_O + e^−^ → * + H_2_ + OH^−^ (Heyrovský step),^[^ ^8^, ^17^ ^]^ shown in **Figure** **5**a. Therefore, the adsorption free energy of water (Δ*G*_H2O*_) of initial catalyst‐water (hydroxylion) state is the decisive descriptor for alkaline HER process.

**Figure 6:**
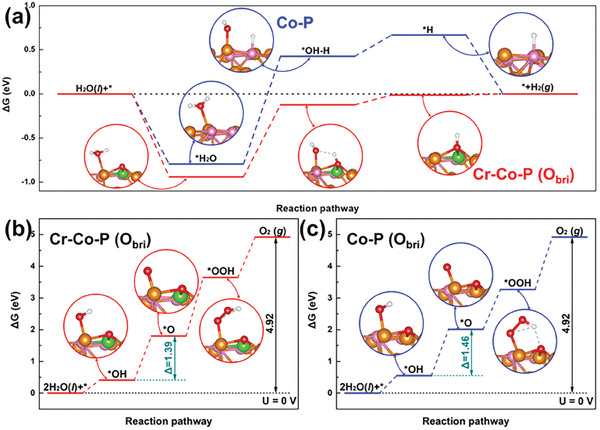
Free energy diagram of a) HER and b,c) OER on the metal centers from the basal plane sites.

As shown in Figure S12b (Supporting Information), the H_2_O molecule was bonded to the outer Co (Connected to another Co through P, denoted as 1) vouched for the absorption active site, where the yellow and mint green colors mean electron gain and electron loss, respectively. Plainly, H atoms are surrounded by a large area of electron depletion, in contrast, P‐Co atom pairs are surrounded by electron enrichment area. Whatever, the H atom tends to be inveigled by O‐Co atom pair rather than P‐Co atom after the introduction of O atom in model, and there even are much more electrons around O‐Cr atom pairs after the introduction of CrO_x_ species, resulting in the enhanced binding interaction between O‐Cr and H, which is beneficial for promoting the dissociation process of water. Apparently, the construction of Co‐O‐Cr atom couple in cobalt phosphide crystals can significantly impact the adsorption state of the catalyst surface, which is also reflected in the changes in the density of site (DOS) and d‐band center (ɛ_d_). As shown by Figure 5c, it is clearly that models with Cr doping have a larger DOS than that of unadulterated Co‐P at the Fermi level (set to 0 eV), suggesting the promoted electronic transmission capability in Cr‐Co‐P, which also verifies the conclusion of EIS test. In addition, the d‐band center of the catalyst shifts away from the fermi level after O‐embedded in C‐P (Δ = 0.04 eV) and Cr‐Co‐P (Δ = 0.05 eV) owing to the strong interaction between oxygen‐bridged cobalt–chromium atomic pair. The downshift d‐band center has been proved to be beneficial to HER process by a large number of investigations by adjusting the adsorption energy of intermediate according to the d‐band theory.^[^ ^8^, ^43^ ^]^ Ultimately, the Co sites in Co‐O‐Cr couple on basal plane sites does have a stronger ability to capture water molecules compared with that in Co‐O‐Co according to the calculated H_2_O adsorption energy in Figure 5d. Herein, Co sites in oxygen‐bridged cobalt–chromium atomic pair provide the best HER catalytic activity on basal plane sites that benefited from its hydrophilicity and oxophilicity. As shown in the free energy landscape (**Figure** **6**a), the dissociation of water is the rate‐limiting step for both Co‐P and Cr‐Co‐P (O_bri_) with the maximum free energy changes, which even exceeds 1 eV. Regardless, even though Cr‐Co‐P (O_bri_) exhibits superior hydrophilicity, its dissociation energy of adsorbed water molecule is still 0.16 eV lower than that of Co‐P, which is suggested by comparing the *OH─H co‐adsorption energy change (∆G_*OH‐H_). Hence, Co‐O‐Cr couple on basal plane sites exhibit significantly enhanced activity to adsorb *H and *OH. In the meantime, the *H adsorption energies for O site in Co‐O‐Cr atom couple and P site in Co‐P‐Co atom couple are compared. Generally, zero is the moderate value for the *H adsorption energy (ΔG_*H_) on the surface. As expected, Co‐O‐Cr atom couple possesses an *H adsorption energy with the closest value to zero, which is commonly believed to be the typical characteristics of the best catalyst.

Subsequently, DFT calculations were further performed to decode the roots of the interfacial effect in OER, in which four‐electron mechanism put forward by Nøskov. Previous studies have pointed out that the strong oxidizing conditions of the OER process can promote in situ self‐reconstruction of the catalyst surface, which will make the surface of phosphide evolve into (oxy)hydroxide.^[^ ^8^, ^18^, ^44^ ^]^ Therefore, O‐embedding catalysts of Co‐P (O_bri_) and Cr‐Co‐P (O_bri_) are selected as models (Figure S13, Supporting Information) according to the model construction strategy proposed by Yan et al.^[^ ^45^ ^]^ Ordinarily, both the low‐spin state (*t_2g_^6^e_g_^0^*) of Co^3+^ and the bridging O^2−^ in CoOOH have the fully occupied *π*‐symmetry (*t_2g_*) d‐orbitals, which will cause the electron–electron repulsion in Co─O─Co unit (seen the schematically explained in Figure 5e) and thereby hinder the electron transfer process between oxidation species and slab.^[^ ^44^, ^46^ ^]^ Based on the XPS results, Cr on the catalyst surface mainly exists in the form of Cr^3+^ ions (*t_2g_^3^e_g_^0^*), in which only three unpaired electrons in the *t_2g_* orbitals are filled in a spin parallel manner, resulting the electron redistribution from Co to Cr in oxygen‐bridged cobalt–chromium atomic pair, which not only relieves the repulsion between O 2p and Co 3d, but also provides theoretical support for the analysis results of XPS. In the end, the electronic structure and orbital changes of catalysts weaken the d–d coulomb interaction of Co sites, leading to the optimization of the interaction between the oxygen‐containing intermediate (*O, *OH, *OOH, etc.) and Co site in Cr‐Co‐P (O_bri_). Figure 6b,c showed the conducted four‐step OER free‐energy diagrams which could reveal the effect of the embedding Cr for OER process. In order to compare and contrast the enhancements, the adsorbed energy difference in the formation of the adsorbed oxygen intermediate during dehydrogenation (difference between ΔG_*OH_ and ΔG_*O_) was selected as the representative, and it was also considered as an indicator and the potential‐determining step (PDS) to describe the catalytic ability for the OER.^[^ ^45^ ^]^ Furthermore, the value of PDS is evidently smaller for Cr‐Co‐P (O_bri_), which verify the trend in the OER testing experiments.

## 3 Conclusion

To summarize, oxygen‐bridged cobalt–chromium unit embedded in cobalt phosphide were successfully prepared via the MOF‐mediated synthetic strategy, showing impressive and easily‐coupled HER and OER catalysts for overall water splitting. Impressively, only an overpotential of 87 and 203 mV were required at 10 mA cm^−2^ for HER and OER, respectively, and the electrolyzer coupled by same Cr‐Co‐P (O_bri_) only need 1.53 V to afford 20 mA cm^−2^ for overall water splitting. DFT investigations demonstrate that the interfacial interaction and electronic structure adjustment enables us to effectively promote the activation of water molecules and modulate energy barrier of intermediates’ conversion. However, this work introduces a promising avenue to create novel bifunctional electrocatalysts toward overall water splitting.

## 4 Experimental Section

See details in the Experimental Section of the Supporting Information.

## Conflict of Interest

The authors declare no conflict of interest.

## Supporting information

Supporting Information

## Data Availability

The data that support the findings of this study are available on request from the corresponding author. The data are not publicly available due to privacy or ethical restrictions.
