# Supplementary material for: Oxygen‐Bridged Cobalt–Chromium Atomic Pair in MOF‐Derived Cobalt Phosphide Networks as Efficient Active Sites Enabling Synergistic Electrocatalytic Water Splitting in Alkaline Media
Source: Adv Sci (Weinh). 2023 Nov 23;11(3):2306678. doi: 10.1002/advs.202306678 (PMC10797420; doi:10.1002/advs.202306678)
Supplement: Supplementary file 1 — Supporting Information [file ADVS-11-2306678-s001.pdf]

ADVANCED SCIENCE
Open Access

# Supporting Information

for *Adv. Sci.*, DOI 10.1002/advs.202306678

Oxygen-Bridged Cobalt–Chromium Atomic Pair in MOF-Derived Cobalt Phosphide Networks as Efficient Active Sites Enabling Synergistic Electrocatalytic Water Splitting in Alkaline Media

*Zepeng Lv*, Huakui Zhang, Chenhui Liu, Shaolong Li, Jianxun Song* and Jilin He*

# Supporting Information

**Oxygen-Bridged Cobalt-Chromium Atomic pair in MOF-Derived Cobalt Phosphide Networks as Efficient Active Sites Enabling Synergistic Electrocatalytic Water Splitting in Alkaline Media**

*Zeping Lv*,#, Huakui Zhang#, Chenhui Liu, Shaolong Li, Jianxun Song*, Jilin He*

## Experimental Section

### *Materials*

KOH (90%), $Co(NO_3)_2 \cdot 6H_2O$ (99%), $Cr(NO_3)_3 \cdot 9H_2O$ (99%), $C_4H_6N_2$ (98%), $NaH_2PO_2$ (98%) and ethanol (99.5%) were purchased from Shanghai TITAN Technology Co., Ltd. (China). All the reagents and chemicals were of analytical grade and used directly without further purification.

### *Preparation of Co-MOF precursor*

The cobalt-based metal-organic framework supported on nickel foam (NF) was prepared according to the previous report.[1] In a typical process, the commercial NF was pre-washed successively with ethanol, deionized water, and ethanol, each for 15 min, to ensure a clean surface. The bare Ni foam was first immersed into 2-methylimidazole aqueous solution (0.4 M, 40mL) overnight and then immersed into the mix solution of $Co^{2+}$ and 2-methylimidazole for another 6 hours. In the end, a uniform purple layer could be observed on Ni foam. The growth mechanism of Co-MOF is therefore believed to be as follows: 2-methylimidazole molecules are initially adsorbed on the Ni foam surface and then coordinate with $Co^{2+}$ ions in the aqueous solution to form ZIF-Co.

***Fabrication of Cr-Co-MOF precursor***

A piece of as-fabricated Co-MOF-LN were immersed into an aqueous ethanol solution (100 mL) containing $Co(NO_3)_2$ and $Cr(NO_3)_3$ and kept stationary to allow an in-situ conversion from Co-MOF to Cr-Co-MOF composite on the surface of NF substrate, where $Cr(NO_3)_3$ accounts for 0~20% of the total number of moles of $Co(NO_3)_2$ and $Cr(NO_3)_3$. After a reaction of 15~45 min under room temperature, the resulting product was washed with deionized water and absolute ethanol for three times, respectively, and then dried at 60 $^oC$ to provide a Cr-Co-MOF sample.

***Preparation of Co-P, Cr-Co- P***

To synthesize Co-P, Cr-Co-P, the corresponding precursors and 800 mg of $NaH_2PO_2$ were added into two crucibles, respectively, and $NaH_2PO_2$ was placed at the upstream side of gas flow. They are then heated for a phosphorization at 300 °C in Ar flowing for 1 h with a heating rate of 2 °C $min^{-1}$. After cooling down in an Ar atmosphere, the Co-P, Cr-Co-P were collected.

***Characterizations***

Field emission scanning electron microscopy (FE-SEM) images were obtained using a FEI Quattro S (ThermoFisher Scientific). TEM images were taken using a FEI Talos F200S (ThermoFisher Scientific) scanning transmission electron microscope with an operating voltage of 200 kV. XPS analysis was carried out using an ESCALAB250Xi system (ThermoFisher Scientific). XRD patterns were obtained from a PANalytical X'Pert Powder using Cu Kα radiation (λ = 1.5406 Å). Precision balance instrument (FA224TC, Lichen Instrument Technology Co., Ltd) was used to weigh the loading mass of different catalysts. Raman spectra were carried out on a Raman spectrometer (HORIBA LabRam HR 800). The nitrogen adsorption-desorption isotherm of the sample was measured on a Quantachrome QuadraSorb Station 3 instrument at 77 K.

***Electrochemical measurements***

Electrochemical measurements were carried out using CHI660E electrochemistry work station in a standard three-electrode system. The working electrode was the Cr-Co-P (and other control samples) with an effective geometric area of 2 $cm^2$. Hg/HgO electrode and graphite rod were used as reference electrode and counter electrode, respectively. All electrochemical data was with 90% *iR*-correction. 1M KOH were used as electrolytes. All measured potentials were converted to a reversible hydrogen electrode (RHE) scale using Nernst equation: E(RHE) = E(Hg/HgO) + 0.098V + 0.059 × pH. For HER testing, the linear sweep voltammetry (LSV) measurements were performed at a scan rate of 5 $mV{\cdot}s^{-1}$. Electrochemical impedance spectroscopy (EIS) measurements were conducted at the frequency range from $10^6$ to $10^{-2}$ Hz in -0.1V. The cyclic voltammetry (CV) was conducted at various scan rates (10-100 $mV{\cdot}s^{-1}$). The electrochemically active surface area (ECSA) was estimated by measuring the capacitive current associated with double-layer charging from scan-rate dependence of CVs in a non-Faradaic region and the scan rates were 10-60 mV $s^{-1}$. The multi-step chronopotentiometric curves were obtained by changing the current densities from -10 and 100 mA $cm^{-2}$. The stability of the catalyst films was assessed by repeated CV scans at 100 $mV{\cdot}s^{-1}$ for 3000 sweeps. For HER processes, the potential ranges were set in 0 V ~ -0.20 V *vs.* RHE.

***Computational studies***

The first principles calculations are performed using the Cambridge Serial Total Energy Package (CASTEP) program code based on the plane-wave pseudo-potential method within the framework of density func-tional theory (DFT).[2] The generalized gradient approximation (GGA) is adopted along with the exchange-correlation function realized by Per-dow–Burke–Emzerhof (PBE).[3] A plane wave basis was set up to an energy cutoff of 500 eV for the surface of all catalysts. The convergence tolerance for the residual force on each atom during structure relaxation was set to 0.03 eV/Å and for the energy difference between two consecutive self-consistent calculations is less than $10^{-5}$ eV, respectively. A sufficiently large vacuum region of 20 Å was used for all the systems to ensure the periodic images were well separated, and the

atoms relax during the geometry optimizations. The calculated minimum distance between the different moiety surface was 3 Å.

The computational hydrogen electrode (CHE) was used to calculate the free energy of each intermediate state. The $H_2O$ absorption energy was calculated using the following equation, $\Delta_{EH2O} = E_{surfH2O} - E_{surf} - E_{H2O}$, where $E_{surfH2O}$ and $E_{surf}$ are the total energies of the surface covered with and without $H_2O$ molecule, $E_{H2O}$ is the energy of free $H_2O$ molecule. The overall HER pathway includes two steps: first, adsorption of hydrogen on the catalytic site (*) from the initial state ($H^+ + e^- + *$), and second, release of the product hydrogen ($1/2H_2$). The total energies of $H^+ + e^-$ and $1/2H_2$ are equal. Therefore, the Gibbs free energy of the adsorption of the intermediate hydrogen on a catalyst ($\Delta G_H$) is the key descriptor of the HER activity of the catalyst and is obtained by equation: $\Delta G=\Delta E+\Delta E_{ZPE}-T\Delta S$, where the $\Delta E$ is the adsorption energy of a specific step, and $\Delta E_{ZPE}$ and $T\Delta S$ are the difference of zero point energy and the entropy between the adsorbed state and the free-standing state, respectively. In addition, to evaluate the OER activity of the materials, we calculate the Gibbs free energy of coordinate elementary steps and overpotential for OER based on the following $4e^-$-mechanism proposed by Norskov for water oxidation.

$$H_2O(l)+* \leftrightharpoons *OH+H^++e^- \qquad \Delta G_1=\Delta G_{*OH}-\Delta G_{H2O(l)}-eU+k_bT\,ln\,a_{H+}$$

$$*OH \leftrightharpoons *O+H^++e^- \qquad \Delta G_2=\Delta G_{*O}-\Delta G_{*OH}-eU+k_bT\,ln\,a_{H+}$$

$$*O+H_2O(l) \leftrightharpoons *OOH+H^++e^- \qquad \Delta G_2=\Delta G_{*OOH}-\Delta G_{*O}-eU+k_bT\,ln\,a_{H+}$$

$$*OOH \leftrightharpoons *+O_2(g) +H^++e^- \qquad \Delta G_2=\Delta G_{O2}-\Delta G_{*OOH}-eU+k_bT\,ln\,a_{H+}$$

Where the symbols "*" donates the active sites on the catalyst, (l) and (g) represents liquid and gas phases. The overpotential $\eta$ is defined in Equation:

$$\eta=-max(\Delta G_1, \Delta G_2, \Delta G_3, \Delta G_4)-1.23\ eV$$

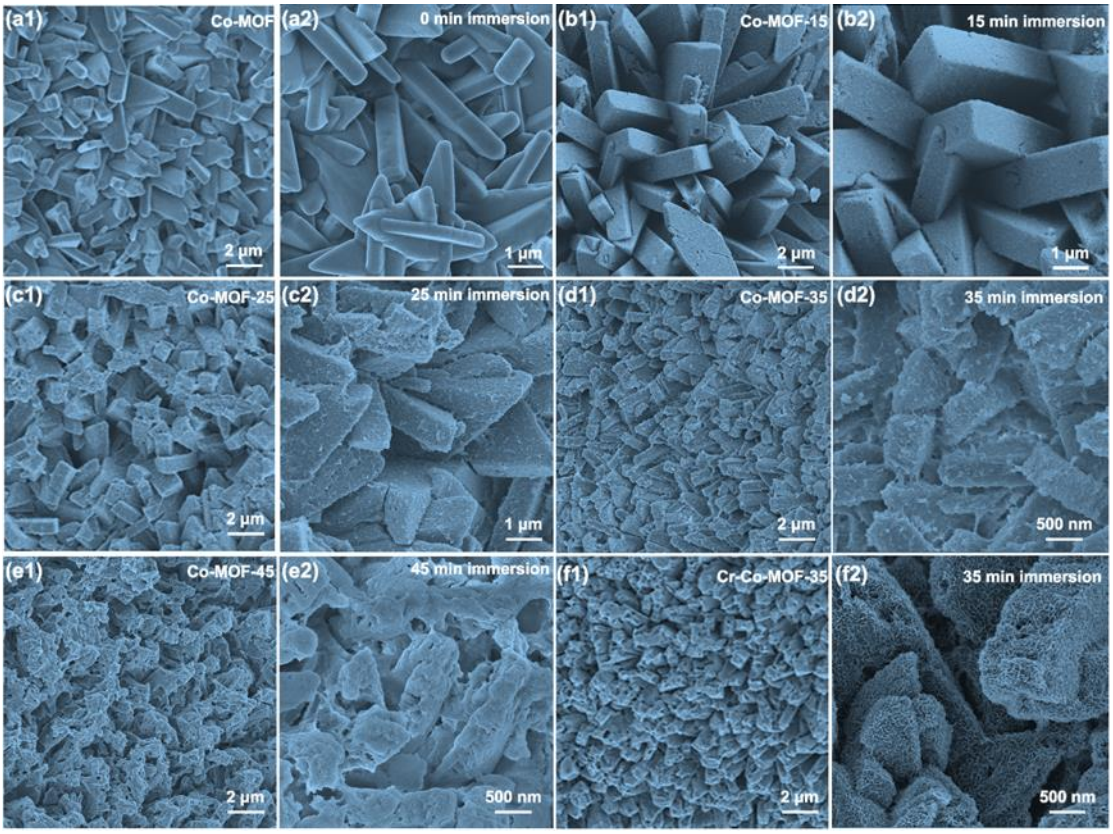


**Figure S1**. SEM images of (a) Co-MOF obtained after the two-step reaction and (b-e) Co-MOF with 15 min, 25min, 35 min, 45 min etching treatment, (f) Cr doped Co-MOF after 35 min etching treatment.

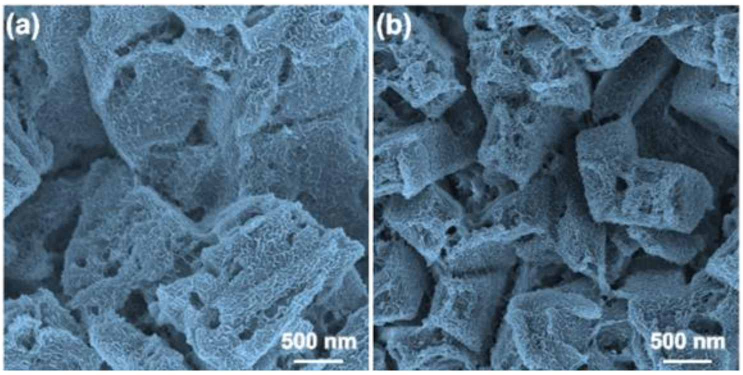


**Figure S2**. SEM images of 5%Cr-Co-P-35.

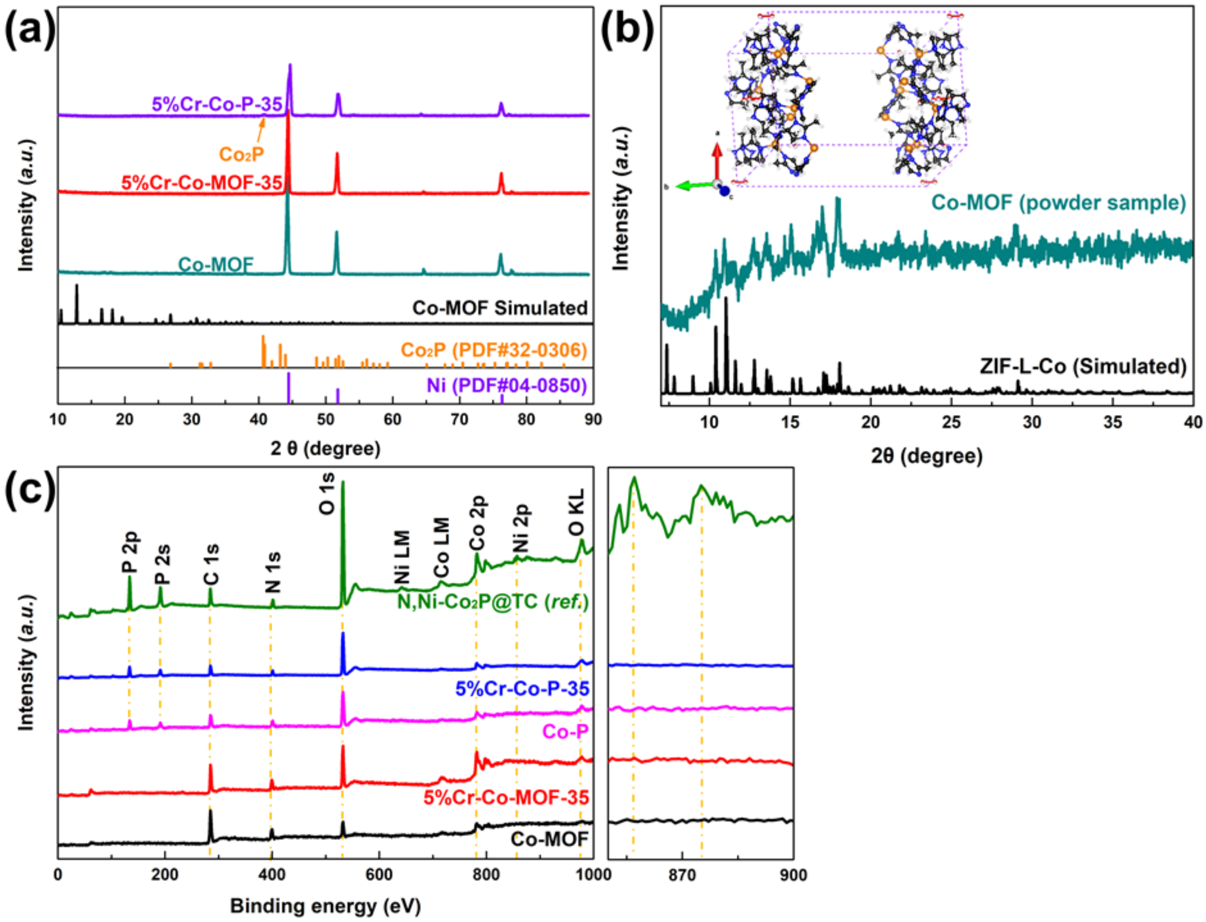


**Figure S3**. (a) XRD patterns of synthesized catalysts, (b) XRD patterns of synthesized Co-MOF peeled off from NF, (c) XPS full spectra of samples (where the result of N,Ni-$Co_2P$@TC was previously reported by us[4]).

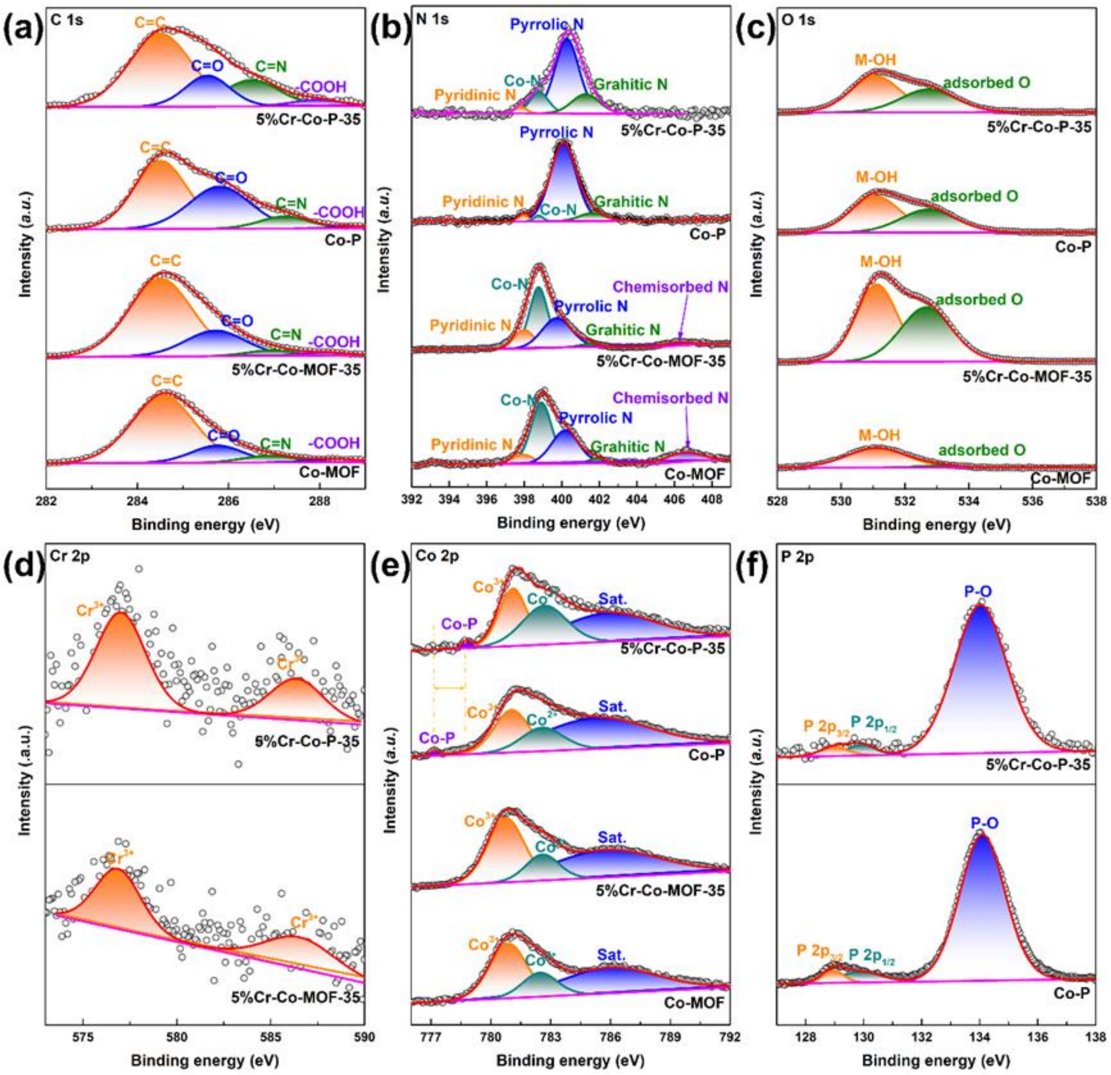


**Figure S4**. High resolution spectra of (a) C 1s, (b) N 1s, (c) O 1s, (d) Cr 2p, (e) Co 2p and (f) P 2p.

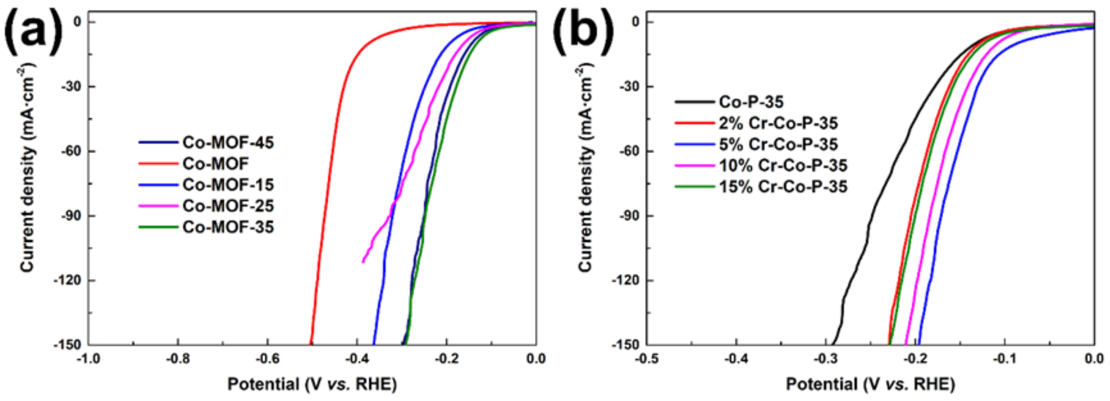


**Figure S5**. HER performance of Co-MOF with (a) different etching times and (b) different Cr doping levels.

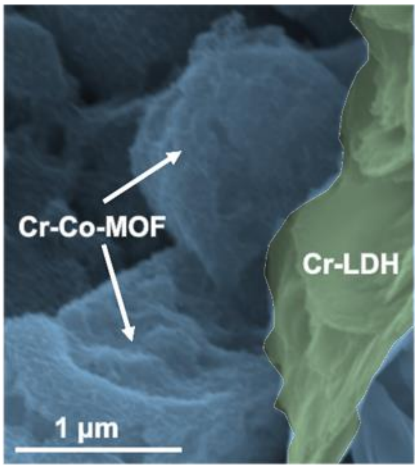


**Fugure S6**. SEM image of 20%Cr-Co-MOF-35.

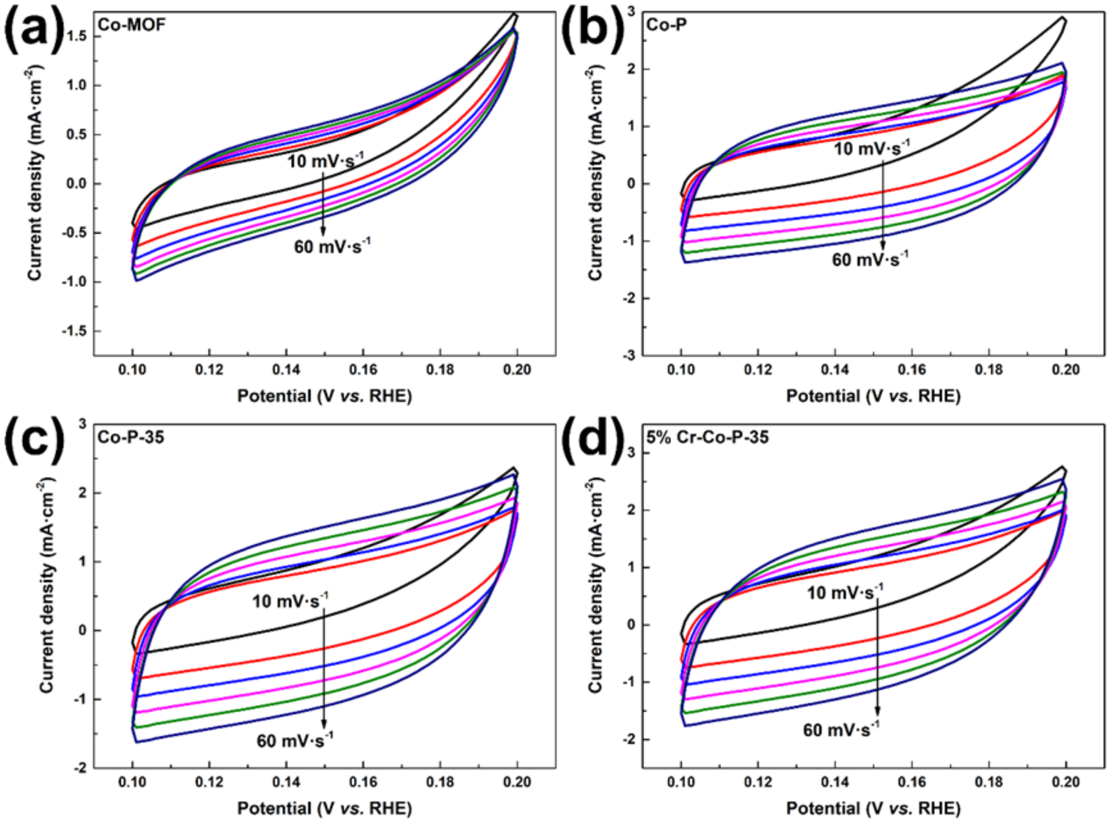


**Figure S7**. (a-d) CV curves of Co-MOF, Co-P, Co-P-35 and 5%Cr-Co-P-35 in the double layer capacitive region at the scan rates from 10 mV· $s^{-1}$ to 60 mV· $s^{-1}$.

**Table S1**. The system resistance (Rs) and charge transfer resistance (Rct) for four samples.

| **Catalysts** | **$R_s$ (Ω)** | **$R_{ct}$ (Ω)** |
|---|---|---|
| Co-MOF | 1.584 | 61.29 |

| | | |
|---|---|---|
| Co-P | 1.607 | 14.32 |
| Co-P-35 | 1.566 | 7.82 |
| 5%Cr-Co-P-35 | 1.599 | 5.67 |

**Table S2**. A summary of the HER performance for reported MOF or MOF-derived electrocatalysts.

| Catalysts | Electrolyte | Overpotential (mV) | | Tafel slope ($mV \cdot dec^{-1}$) |
|---|---|---|---|---|
| | | $\eta_{10}$ | $\eta_{100}$ | |
| CuCo-CAT/CC[5] | 1 M KOH | 52 | ~215 | 64.2 |
| *h*-$Co_{0.34}Fe_{0.33}Ni_{0.33}$-LDH[6] | 1 M KOH | 71 | ~159 | 83 |
| This work | 1 M KOH | 87 | 176 | 81.5 |
| CuSNC@$MoS_2$-Pt[7] | 1 M KOH | 102.6 | ~199 | 55.7 |
| $Co_2Ni_1N$[8] | 1 M KOH | 102.6 | ~261 | 60.17 |
| FeCoMnNi-MOF-74/NF[9] | 1 M KOH | 108 | ~221 | 72.89 |
| $Ti_2NT_x$@MOF-CoP[10] | 1 M KOH | 112 | ~247 | 67.1 |
| CoP/NCNs[11] | 1 M KOH | 118 | ~183 | 59 |
| MOF-V-$Ni_3S_2$/NF[12] | 1 M KOH | 118.1 | ~350 | 113.2 |
| CoS@CoNi-LDH/CC[13] | 1 M KOH | 124 | ~275 | 89 |
| $NiFe_2O_4$@MOF-74[14] | 1 M KOH | 403 | ~544 | 106 |

where the overpotential at -20 $mA \cdot cm^{-2}$ is estimated based on the polarization curves in the literature.

**Table S3**. A summary of the OER performance for reported MOF or MOF-derived electrocatalysts.

| Catalysts | Electrolyte | Overpotential $\eta_{10}$ (mV) | Tafel slope ($mV \cdot dec^{-1}$) |
|---|---|---|---|
| Co-LDH@ZIF-67[15] | 1 M KOH | 187 | 59 |
| *h*-$Co_{0.34}Fe_{0.33}Ni_{0.33}$-LDH[6] | 1 M KOH | 195 | 53 |
| This work | 1 M KOH | 203 | 82.9 |

| | | | |
|---|---|---|---|
| D-Ni-MOF[16] | 1 M KOH | 219 | 48.2 |
| D-U-CoNi-OH[1] | 1 M KOH | 228 | 57 |
| NiFeP-MOF[17] | 1 M KOH | 233 | - |
| NFN-MOF/NF[18] | 1 M KOH | 240 | 58.8 |
| VFe-MOF@NF[19] | 1 M KOH | 246 | 42.61 |
| $Ni_{1.85}Co_{0.925}Fe_{0.075}$-MOF-NF[20] | 1 M KOH | 257 | 41.3 |
| $Co_3O_4$@Co-MOF-12[21] | 1 M KOH | 277 | 79 |
| $Co_2P$/CoNPC[22] | 1 M KOH | 326 | 72.6 |

**Table S4.** A summary of the overall water splitting performance for reported MOF or MOF-derived electrocatalysts.

| **Catalysts** | **Electrolyte** | **Overpotential $\eta_{20}$ (V)** |
|---|---|---|
| *h*-$Co_{0.34}Fe_{0.33}Ni_{0.33}$-LDH\|\|*h*-$Co_{0.34}Fe_{0.33}Ni_{0.33}$-LDH[6] | 1 M KOH | ~1.52 |
| This work | 1 M KOH | ~1.53 |
| D-Ni-MOF\|\|D-Ni-MOF[16] | 1 M KOH | ~1.53 |
| NiFeP-MOF\|\|NiFeP-MOF[17] | 1 M KOH | ~1.57 |
| NFN-MOF/NF\|\|NFN-MOF/NF[18] | 1 M KOH | ~1.61 |
| CoP-$Ni_5P_4$/NF\|\| CoP-$Ni_5P_4$/NF[23] | 1 M KOH | ~1.63 |
| MOF-V-$Ni_3S_2$/NF\|\|MOF-V-$Ni_3S_2$/NF[12] | 1 M KOH | ~1.65 |
| $Co_3O_4$@Co-MOF-12\|\|$Co_3O_4$@Co-MOF-12[21] | 1 M KOH | ~1.66 |
| VFe-MOF@NF\|\|VFe-MOF@NF[19] | 1 M KOH | ~1.70 |
| FeCoMnNi-MOF-74/NF\|\|FeCoMnNi-MOF-74/NF[9] | 1 M KOH | ~1.72 |
| $Co_2P$/CoNPC\|\|$Co_2P$/CoNPC[22] | 1 M KOH | ~1.73 |

where the overpotential at 20 mA· cm$^{-2}$ is estimated based on the polarization curves in the literature.

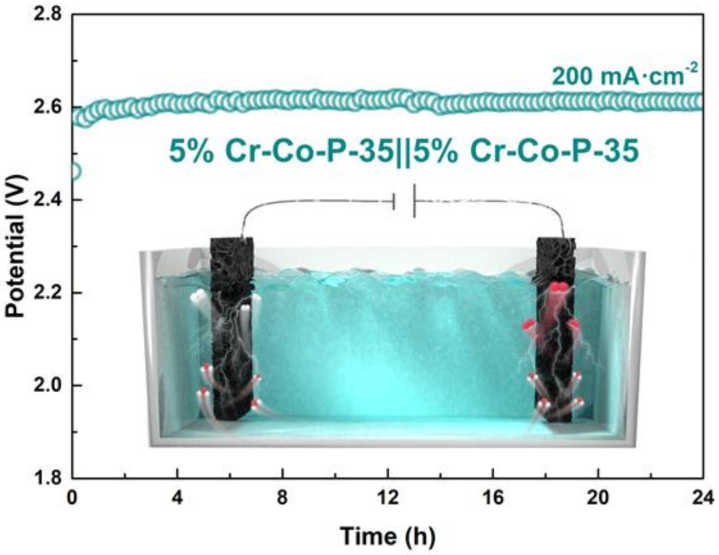


**Figure S8**. The chronopotentiometric curve of the 5%Cr-Co-P-35||5%Cr-Co-P-35 tested at a constant current density of 200 mA cm$^{-2}$ for 24 h

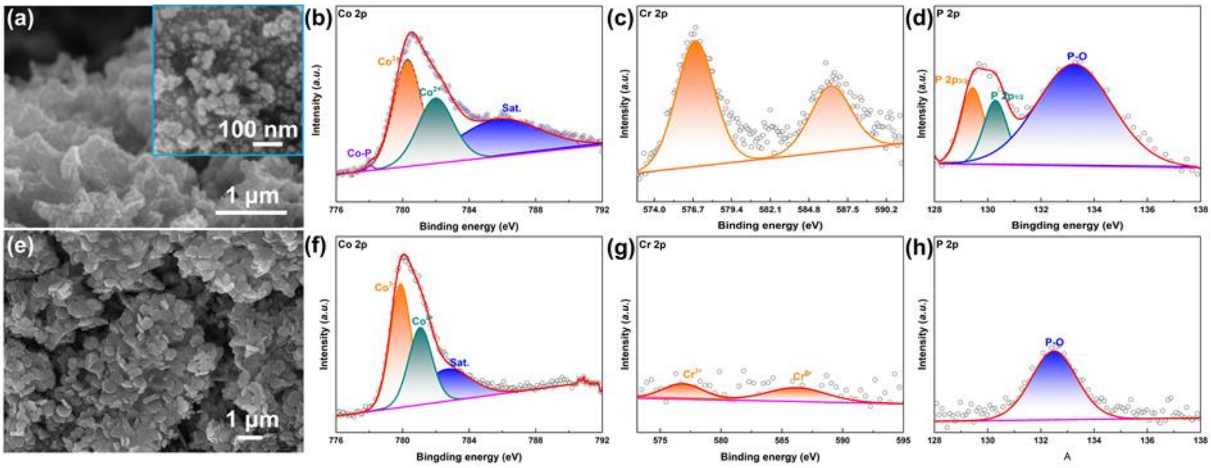


**Figure S9**. SEM images and high-resolution XPS spectra of 5%Cr-Co-P-35||5%Cr-Co-P-35 afteroverall water splitting durability tests, where (a-d) are the test results of the cathode and (e-h) are the test results of the anode.

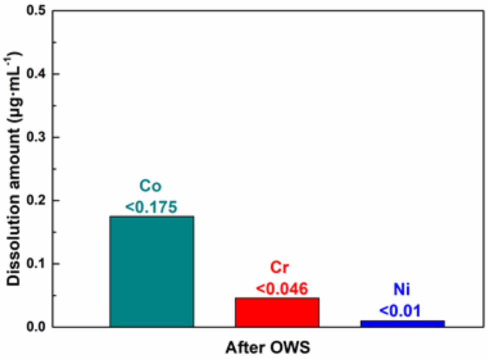


**Figure S10**. Dissolution concentration of Co, Cr and Ni in the electrolyte after OWS catalysis for 24 h (analyzed using ICP-OES).

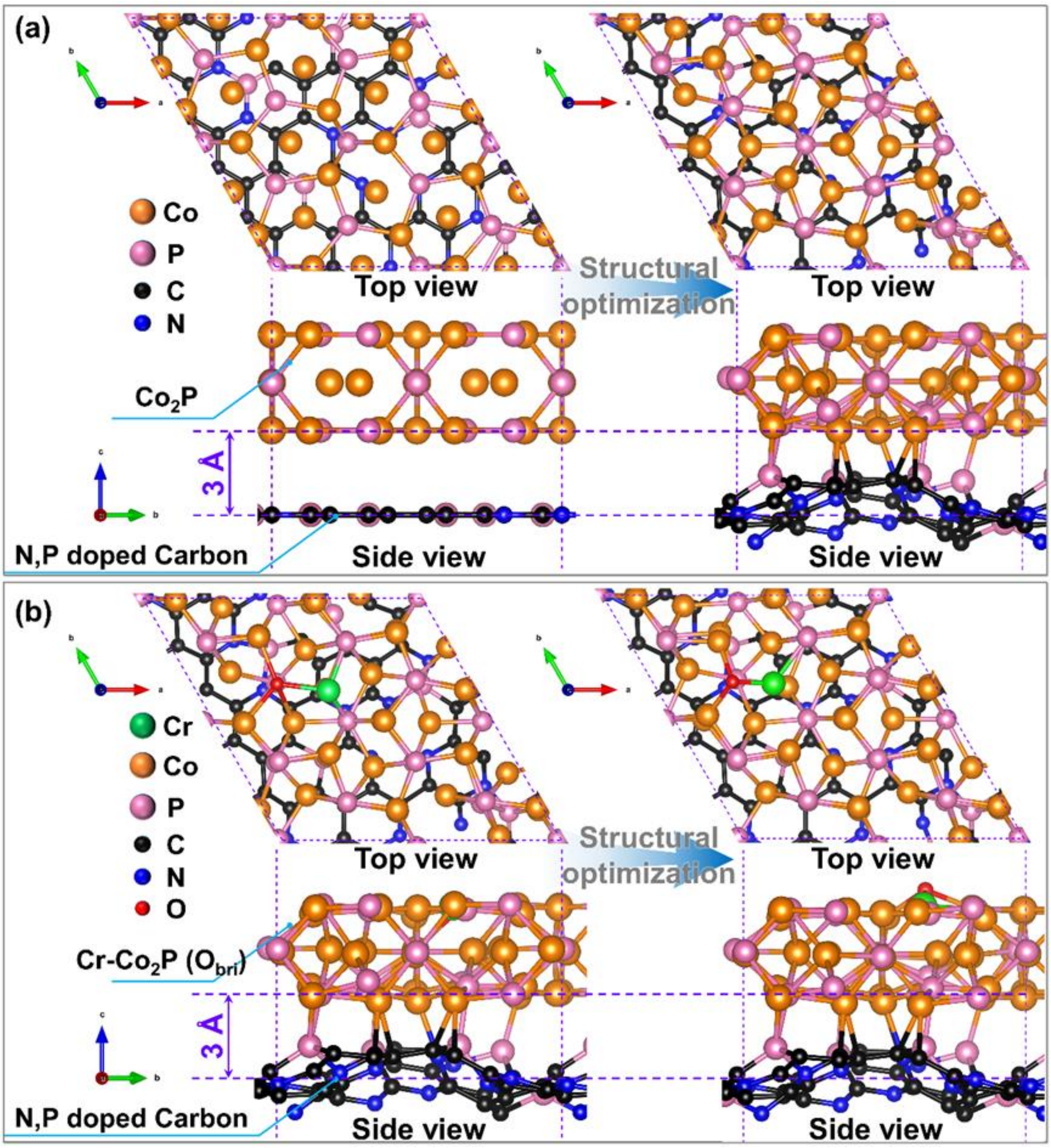


**Figure S11.** The side view and top view of the optimized structure of (a) Co-P and (b) Cr-Co-P ($O_{bri}$) (Substitution for Co-P site with Cr-O atoms on the outermost surface of Co-P).

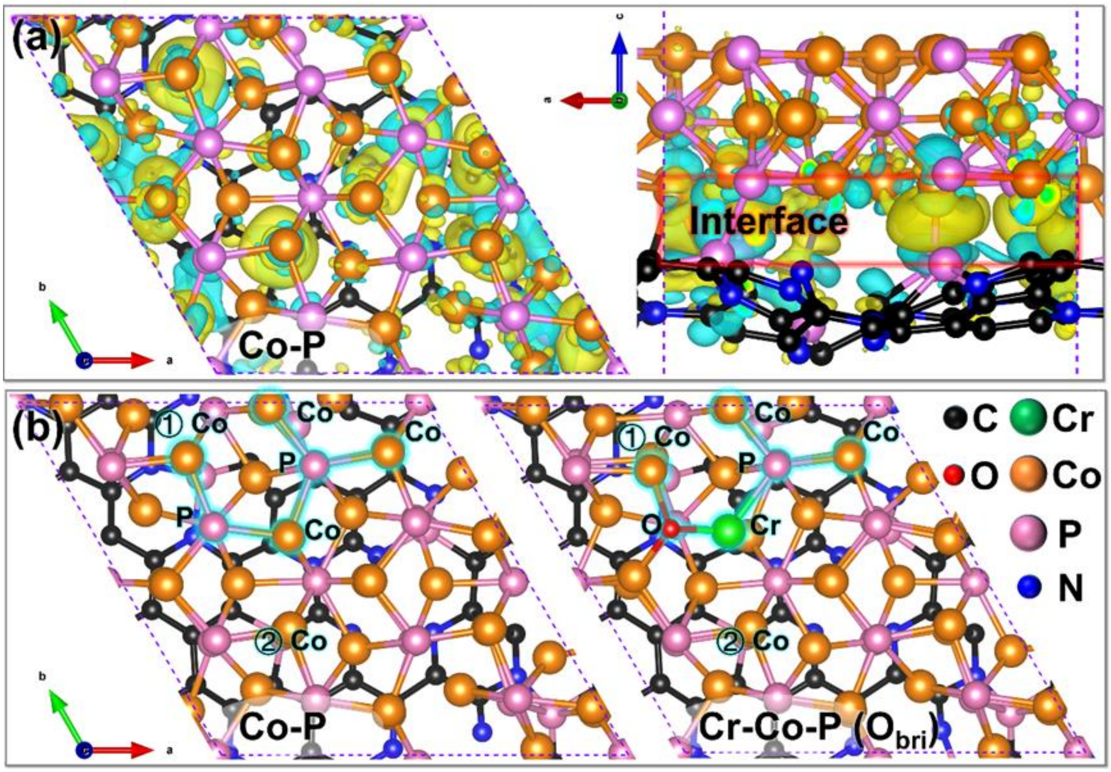


**Figure S12.** (a) Differential charge density distributions of Co-P (Isosurface value: 0.002 e/Bohr$^3$), (b) selection of adsorption active sites in computational models.

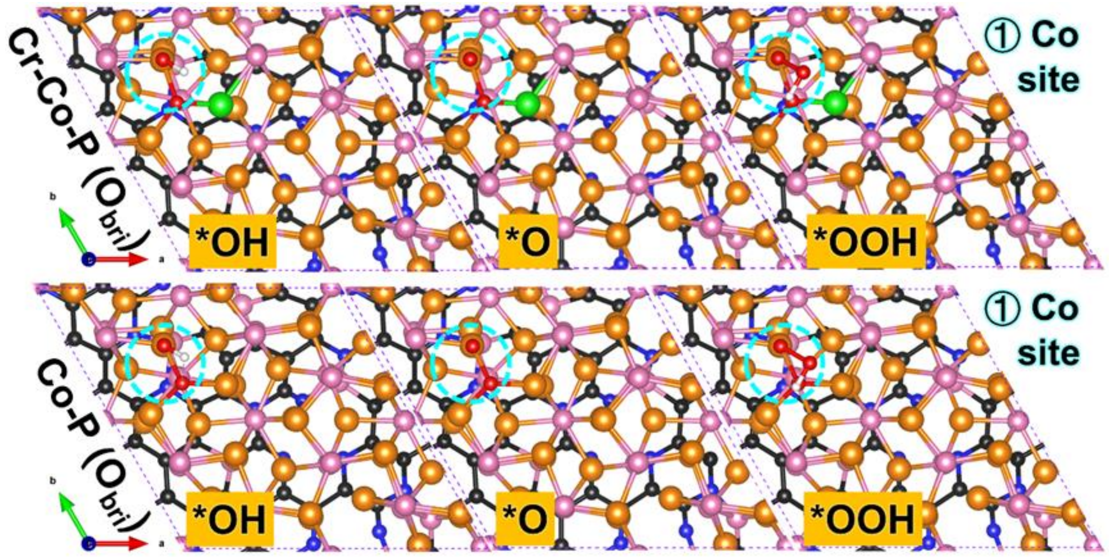


**Figure S13.** Adsorption configurations of *M-OH, *M-O and *M-OOH on Co centers from the basal plane of Cr-Co-P ($O_{bri}$) and Co-P ($O_{bri}$).

References


[1] B. Zhang, Z. Qi, Z. Wu, Y. H. Lui, T. Kim, X. Tang, L. Zhou, W. Huang, S. Hu, *ACS Energy Letters* **2019**, *4*, 328.

[2] S. J. Clark, M. D. Segallii, C. J. Pickardii, P. J. Hasnipiii, M. I. J. Probertiv, *Zeitschrift für Kristallographie - Crystalline Materials* **2005**, *220*.

[3] M. Ernzerhof, G. E. Scuseria, *Journal of Chemical Physics* **1999**, *110*, 5029.

[4] Z. Lv, J. Fei, Y. You, X. Lv, Q. Li, J. Dang, *Journal of Materials Science & Technology* **2023**, *147*, 207.
[5] B. Geng, F. Yan, X. Zhang, Y. He, C. Zhu, S. Chou, X. Zhang, Y. Chen, *Advanced Materials* **2021**, *33*, 2106781.
[6] H. Sun, L. Chen, Y. Lian, W. Yang, L. Lin, Y. Chen, J. Xu, D. Wang, X. Yang, M. H. Rümmerli, J. Guo, J. Zhong, Z. Deng, Y. Jiao, Y. Peng, S. Qiao, *Advanced Materials* **2020**, *32*, 2006784.
[7] J. Rong, G. Zhu, W. Ryan Osterloh, Y. Fang, Z. Ou, F. Qiu, K. M. Kadish, *Chemical Engineering Journal* **2021**, *412*, 127556.
[8] X. Feng, H. Wang, X. Bo, L. Guo, *ACS Applied Materials & Interfaces* **2019**, *11*, 8018.
[9] M. Zhang, W. Xu, T. Li, H. Zhu, Y. Zheng, *Inorganic Chemistry* **2020**, *59*, 15467.
[10] H. Zong, R. Qi, K. Yu, Z. Zhu, *Electrochimica Acta* **2021**, *393*, 139068.
[11] Y. Li, Y. Zou, Y. Bai, X. Zhang, G. Wang, X. Huang, D. Chen, *Journal of Colloid and Interface Science* **2021**, *600*, 872.
[12] W. Dong, H. Zhou, B. Mao, Z. Zhang, Y. Liu, Y. Liu, F. Li, D. Zhang, D. Zhang, W. Shi, *International Journal of Hydrogen Energy* **2021**, *46*, 10773.
[13] K. Ao, Q. Wei, W. A. Daoud, *ACS Applied Materials & Interfaces* **2020**, *12*, 33595.
[14] H. Zheng, H. Chen, Y. Wang, P. Gao, X. Liu, E. V. Rebrov, *ACS Applied Materials & Interfaces* **2020**, *12*, 45987.
[15] Z. Li, X. Zhang, Y. Kang, C. C. Yu, Y. Wen, M. Hu, D. Meng, W. Song, Y. Yang, *Advanced Science* **2021**, *8*, 2002631.
[16] J. Zhou, Y. Dou, X. Wu, A. Zhou, L. Shu, J. Li, *Small* **2020**, *16*, 1906564.
[17] X. Xu, T. Wang, C. Zhao, Z. Huang, M. Zheng, R. Jia, Y. Liu, *Microporous and Mesoporous Materials* **2021**, *312*, 110760.
[18] D. Senthil Raja, X. Chuah, S. Lu, *Advanced Energy Materials* **2018**, *8*, 1801065.
[19] L. Han, J. Xu, Y. Huang, W. Dong, X. Jia, *Chinese Chemical Letters* **2021**, *32*, 2263.
[20] Q. Qian, Y. Li, Y. Liu, L. Yu, G. Zhang, *Advanced Materials* **2019**, *31*, 1901139.
[21] S. Zheng, X. Guo, H. Xue, K. Pan, C. Liu, H. Pang, *Chemical Communications* **2019**, *55*, 10904.
[22] H. Liu, J. Guan, S. Yang, Y. Yu, R. Shao, Z. Zhang, M. Dou, F. Wang, Q. Xu, *Advanced Materials* **2020**, *32*, 2003649.
[23] J. Jiang, G. Xu, Y. Li, C. Wang, L. Zhang, *Journal of Materials Chemistry A* **2023**, *11*, 1801.
